# Genetic analysis of spinal dysraphism with a hamartomatous growth (appendix) of the spinal cord: a case series

**DOI:** 10.1186/s12883-020-01710-7

**Published:** 2020-04-06

**Authors:** Ronald H. M. A. Bartels, J. Andre Grotenhuis, Alexander P. A. Stegmann, Han Brunner

**Affiliations:** 1 Department of Neurosurgery, Radboud University Medical Center, Geert Grote Plein-Zuid 10, 6525 GA Nijmegen, the Netherlands; 2 Department of Clinical Genetics, Maastricht University Medical Center, Maastricht, the Netherlands; 3 Department of Clinical Genomics, Radboud University Medical Center, Geert Grote Plein-Zuid 10, 6525 GA Nijmegen, the Netherlands

**Keywords:** Case series, Abnormal development, Genetic analysis, Spinal cord

## Abstract

**Background:**

Spinal dysraphism with a hamartomatous growth (appendix) of the spinal cord is better known as herniated spinal cord. There are many arguments in favour of considering it a developmental defect. From this point of view, it is a type of neural tube disorder. Neural tube disorders can be caused by multiple factors, including a genetic factor. A common genetic defect in patients with a spinal dysraphism with a hamartomatous growth of the spinal cord is sought for.

**Case presentation:**

In two patients with a symptomatic lesion and referred to an academic hospital a genetic analysis was performed after informed consent. Whole-exome analysis was performed.

**:** Whole-exome analysis did not result in identification of a clinically relevant genetic variant.

**Conclusions:**

This the first study to investigate the genetic contribution to spinal dysraphism with a hamartomatous growth (appendix) of the spinal cord. We could not establish a genetic cause for this entity. This conclusion cannot be definitive due to the small sample size. However, the incidental occurrence, the lack of reports of inheritance of this disorder and the absence of contribution to syndromal disorders favours a defect of normal development of the spinal cord.

## Background

Spinal dysraphism with a hamartomatous growth is also known as idiopathic ventral herniation of the spinal cord [1]. It is a very rare entity with a low prevalence. Up until the end of September 2015, 259 patients have been reported. In 2019 Groen et al. performed a meta-analysis on 246 patients regarding surgical treatment and postoperative course [2]. Due to an increased access to magnetic resonance imaging (MRI), more patients will be diagnosed spinal dysraphism with a hamartomatous growth describes a rare entity in which the spinal cord is tethered through a defect in the dura. Originally, it was thought that this configuration of the spinal cord was due to a herniation of the spinal cord, an active process. However, this mechanism is not plausible, since a significant force to the neuronal tissue is mandatory to cause an extrusion of spinal cord tissue. This action will almost certainly contribute to a more or less acute loss of function of the spinal cord.

It was recently hypothesized that the abnormality was not the result of an active herniation of normal functioning spinal cord tissue, but due to a developmental abnormality. This was supported by histopathological analysis [3]. A recent report on the histopathologic evaluation of ventral dura surrounding the ventral defect of the spinal cord might support this hypothesis. A loose arrangement of collagen fibres, edematous changes, minor inflammatory cell infiltration and angiogenesis were found [4]. A chronic slight irritation of the surrounding tissue by a tethered spinal cord might result in the described findings. Therefore, idiopathic herniation of the spinal cord is a misnomer, and it was suggested to replace it by a spinal dysraphism with a hamartomatous growth (appendix). Patients with this entity often present with gradual progressive symptoms often like a Brown-Séquard syndrome or with an acute neurologic syndrome, for example after a minor fall. The symptoms seem to arise because of the tethering effect on the spinal cord.

The abnormality can be considered as a disorder of neural tube formation, the most common congenital abnormality with varying prevalence dependent upon race and country. Disorders of neural tube formation are related to genetic but also to environmental factors [5, 6]. This holds also true for an appendix of the spinal cord. In order to investigate whether a genetic variation contributed to the formation of an appendix of the spinal cord, gene-sequence analysis was performed in symptomatic patients.

## Case presentation

**Fig. 1:**
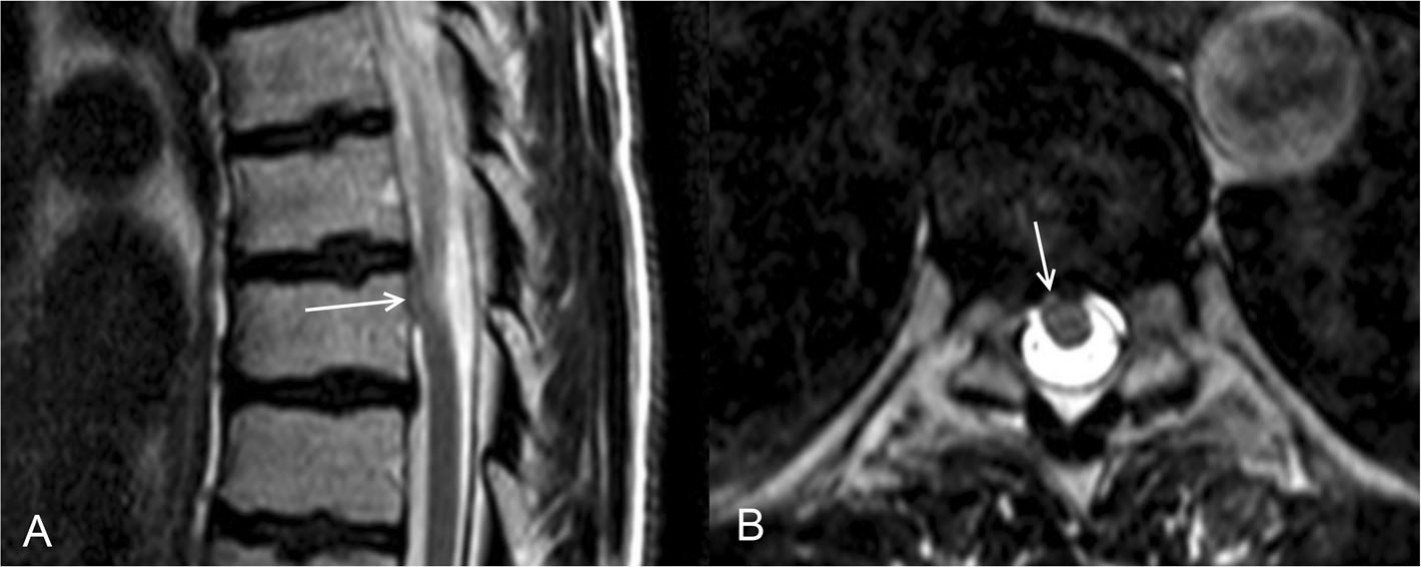
**a** Sagittal T2 weighted MRI of patient 2 showing a ventral displacement of the spinal cord due to spinal dysraphism with a hamartomatous growth (arrow). On the axial view (**b**), the defect is also shown (arrow) as a spinal dysraphism with a hamartomatous growth

Spinal dysraphism with a hamartomatous growth (appendix) of the spinal cord might be the expression of a genetic alteration. Two patients presenting with signs and symptoms due to an appendix of the spinal cord were included (Table 1): a 59-year old female with a lesion at th8 who has been successfully been operated upon, and a 37-year old male with a lesion at th7 (Fig. 1) who refused surgery at this moment. After extensive genetic counselling, the patients gave informed consent to participate. These patients were not consecutive since only two patients out of four visiting our academic hospital from 2015 till 2017 were willing to participate.

**Table 1:** Characteristics of two patients diagnosed with spinal dysraphism with a hamartomatous growth (appendix) of the spinal cord

	Patient 1	Patient 2 (Fig. 1)
Gender	Female	Male
Age (years)	59	37
Level	th8	th7
Previous history	Hypertension	Car accident 10 years prior without severe injuries
Medical history	Pregabalin, Valsartan	Amitriptyline, Omeprazole
Family history	Uneventful	Uneventful
Psychosocial history	Recreational alcoholic consumption, non-smoker, housewife, married	Recreational alcoholic consumption, smoker, unemployed, married
Duration of symptoms	1 year	10 years, soon after the accident
Course until presentation	Progressive	Somewhat gradually
Clinical symptoms	Loss of sensitivity to touch, pain, and temperature in the left leg, buttock, and side of the torso below the ribcage; less strength in the left leg	Dull pain initially only on the left side and discrete loss of sensitivity in a 6-in. band around the ribcage; later, sensitivity was also altered in the left leg; strength remained normal
Physical exam	Vital and gnostic sensibility loss distal to th10 on the left side, discrete paresis of the left biceps femoris muscle (MRC grade 4/5), and symmetrical hyporeflexia in the arms and legs, except for a positive Babinski’s sign on both sides	Sensibility loss distal to th12 on the left side; motor strength and reflexes were normal
Treatment	Surgical exploration and untethering of the spinal cord	Refused surgery
Course	Two years postoperatively: no progression of clinical signs and symptoms after an uneventful postoperative course	Remained very afraid of surgery three years after his first presentation although the clinical signs and symptoms slowly but gradually had worsened

Routine diagnostic exome sequencing and variant calling was performed as described previously [7]. Briefly, the exome was captured using the Agilent SureSelectXT Human All Exon v5 library prep kit (Agilent Technologies, Santa Clara, CA, USA). Exome libraries were sequenced on an Illumina HiSeq 4000 instrument (Illumina, San Diego, CA, USA) with 101 bp paired-end reads at a median coverage of 75× at the BGI Europe facilities (BGI, Copenhagen, Denmark). Sequence reads were aligned to the hg19 reference genome using Burrows-Wheeler Alignment version 0.5.9-r16.14. Variants were called using the unified genotyper Genome Analysis Toolkit, version 3.2–2, and annotated in a custom-built annotation pipeline developed for diagnostics. Variant interpretation was done adhering to professional guidelines [8].

Routine diagnostic whole-exome sequencing using genomic DNA of each patient was initially performed for defined gene panels consisting of genes associated with Mendelian-inherited disorders and neuropathies (for gene panels content, see https://order.radboudumc.nl/en/genetics). In neither patient did this result in the identification of a clinically relevant variant. Subsequently, the entire exome outside the gene panels was interrogated and compared between the two patients. A dual strategy was explored, in search of a biologically plausible candidate gene in which both patients could be shown to carry a potentially significant variant, be it shared by both patients or be it distinct clinically relevant variants in the same gene in each patient. This approach did not result in the identification of a candidate gene.

## Discussions and conclusions

Several causes occurring after complete development of the spinal cord, e.g. trauma, herniated disc, etc. have been suggested for this anomaly. As has been reported, many arguments contradict this hypothesis [1].

Since the location of this entity is relatively constant in most patients with a late presentation and mild neurologic symptoms, a defect in the normal development of the spinal cord, resulting in a tethered spinal cord, was suggested. This defect is always located in the anterolateral thoracic part of the spinal cord between the intumescentia cervicalis and intumescentia lumbalis [1]. In an earlier report [1], the hypothesized mechanism that resulted in a defect of neurulation between 30 and 60 days of gestational age is extensively described. Other authors have also supposed that the defect was congenital [9–11].

As there are multiple factors that can explain the origin of other developmental disorders of the spinal cord, including a genetic factor, a genetic origin for this entity might be possible. This is the first study examining a genetic cause for a rare defect of the neural tube: spinal dysraphism with a hamartomatous growth.

In these two clinically highly similar cases of thoracic spinal dysraphism with a hamartomatous growth, a genetic cause could not be readily identified using routine diagnostic variant filtering and selection when interrogating the entire exome. However, this does not exclude the existence of an underlying genetic cause, since only two cases were compared with a search strategy at diagnostic data probing level. Deeper interrogation of the genome, including less stringently filtered raw sequencing data in a research setting and preferably in a larger cohort size, is needed for a potentially more successful approach.

However, this finding might be in accordance with the presentation of the spinal dysraphism with a hamartomatous growth. In patients, this is the sole lesion. Concomitant abnormalities in other organ systems have not been reported. Furthermore, neither have positive family histories for this entity been described.

The regional differentiation of the cells within the ventral spinal cord is dependent upon local signals from midline cells of the notochord, floor plate and somatic mesoderm [12, 13]. Local transcriptional processes by homeodomain proteins induce cellular subtypes [14, 15]. The development of a spinal dysraphism with a hamartomatous growth could be the consequence of an incidental mismatch in these delicately organized processes.

The sample size is small. Due to the very low incidence, very few cases are seen. We have asked more patients to take part in the study; however, after genetic counselling, they refused informed consent. It should be emphasized that whole-exome sequencing has been proven to be an effective and a time-saving procedure to facilitate the identification of genetic causes of very rare diseases [16, 17].

We were aware that in this sample size the chance of a positive finding was small, but in the past, investigations with this technique in small samples have been positive [18]. Despite this consideration we were convinced that it was worthwhile to investigate.

In conclusion, we could not detect a genetic cause for a spinal dysraphism with a hamartomatous growth. It might be assumed that an incidental mismatch in the transcriptional processes contributes to a defect of normal development of the spinal cord.

## Data Availability

The data that support the findings of this study are available from the corresponding author upon reasonable request.

## Abbreviation

MRI: magnetic resonance imaging
